# Some Suggestions from PK/PD Principles to Contain Resistance in the Clinical Setting—Focus on ICU Patients and Gram-Negative Strains

**DOI:** 10.3390/antibiotics9100676

**Published:** 2020-10-06

**Authors:** Chiara Adembri, Andrea Novelli, Stefania Nobili

**Affiliations:** 1 Department of Health Sciences, Section of Anesthesiology and IC, University of Florence, 50134 Firenze, Italy; chiara.adembri@unifi.it; 2 Department of Health Sciences, Section of Clinical Pharmacology and Oncology, University of Florence, 50139 Firenze, Italy; andrea.novelli@unifi.it

**Keywords:** PK/PD, Monte Carlo Simulation, MDR, gram-negative bacteria, resistance, intensive care unit patients, TDM, antimicrobial stewardship

## Abstract

The containment of the phenomenon of resistance towards antimicrobials is a priority, especially in preserving molecules acting against Gram-negative pathogens, which represent the isolates more frequently found in the fragile population of patients admitted to Intensive Care Units. Antimicrobial therapy aims to prevent resistance through several actions, which are collectively known as “antimicrobial stewardship”, to be taken together, including the application of pharmacokinetic/pharmacodynamic (PK/PD) principles. PK/PD application has been shown to prevent the emergence of resistance in numerous experimental studies, although a straight translation to the clinical setting is not possible. Individualized antibiotic dosing and duration should be pursued in all patients, and even more especially when treating intensive care unit (ICU) septic patients in whom optimal exposure is both difficult to achieve and necessary. In this review, we report on the available data that support the application of PK/PD parameters to contain the development of resistance and we give some practical suggestions that can help to translate the benefit of PK/PD application to the bedside.

## 1. Introduction

The “correct” use of antimicrobials is imperative, not only in obtaining clinical cures and avoiding toxicity, but also to limit the development of resistance [1]. This is particularly true when treating Gram-negative bacteria, which represent nowadays the most frequent pathogens involved in bacteremia and in other hospital-related infections, especially in critically ill patients admitted to intensive care units (ICU) [2,3]. Most of these Gram-negative pathogens show an increasing antimicrobial resistance profile to commonly-used molecules [4]. Therefore, although new molecules have recently been released for clinical use [5], without a global approach called “antimicrobial stewardship” (AMS) [6], these new drugs will lose their efficacy in a short time. The application of pharmacokinetic/pharmacodynamic (PK/PD) principles to antibiotic therapy represents a key point of AMS.

This review summarizes the correct use of antimicrobials, in order to contain resistance, with particular attention to PK/PD principles as part of AMS, exclusively applied to old and new molecules active against Gram-negative strains, and to critically ill septic patients. Data for PK/PD and resistance are mainly available from experimental studies. Suggestions for the translation of PK/PD principles to the clinical setting in ICU patients are given as surrogate. Since clinicians frequently lack detailed knowledge about the application of PK principles towards individualized treatments, we want to offer a practical approach to this aim and to consolidate any gaps in the current literature. The factors affecting dosing (i.e., renal function) and duration (biomarker-guided) of therapy and suggestions for the individualization and optimization of antimicrobial therapy by using bedside therapeutic drug monitoring (TDM), have been examined in this review.

PubMed and Cochrane Library were searched for peer-reviewed articles published in English until July 2020. The reference lists of the articles emerged were also searched to identify additional relevant studies. Terms searched were antimicrobials, pharmacokinetics/pharmacodynamics, antimicrobial resistance, critically ill, intensive care unit, antimicrobial stewardship, Monte Carlo Simulation, Therapeutic Drug Monitoring, Gram-negative strains, MDR.

## 2. Antimicrobial Stewardship and PK/PD Principles

In the near future, there will possibly be non-pharmacological tools and/or non-antibiotic therapies to fight bacterial diseases. Immunotherapy is the most advanced alternative approach with a number of antibodies, including bezlotuxumab targeting *Clostridium difficile*, already approved by FDA [7]. Another strategy is represented by the exploitation of bacteriophages genetically modified that may be engineered to produce bacterial-biofilm-degrading enzymes upon infection, to overexpress genes able to repress bacterial DNA repair systems, to deliver genes able to make pathogens more susceptible to antibiotics or to express antimicrobial peptides and toxins. Currently a bacteriophage formulation received FDA approval limited to food industry [8]. The most relevant examples of other approaches potentially suitable to treat bacterial infections are the following: Interference with “Quorum sensing”—the bacterial communication system for coordinating behavior and production of virulence factors—and inhibition of control gene expression; lysins, such as peptidoglycan degrading proteins endolysins that can be engineered and are currently in clinical trials; Synthetic Mimics of Antimicrobial Peptides (SMAMPs) that, in addition to show some activity against bacteria, are also able to resensitize drug-resistant bacteria to traditional antibiotics; Innate Defense Regulatory (IDR) peptides that although endowed only by immune modulating activity, have been shown to protect animals by death due to severe bacterial infections [9]. At present, however, antibiotics represent the backbone of therapy against bacterial infections and AMS helps physicians to use antibiotics judiciously. There are several definitions of AMS released by international scientific institutions and societies; the European Society of Clinical Microbiology and Infectious Diseases (ESCMID) provided the following: “*A coherent set of actions which promote using antimicrobials responsibly*” [10] and the National Institute for Health and Care Excellence (NICE) the following “*An organisational or healthcare-system-wide approach to promoting and monitoring judicious use of antimicrobials to preserve their future effectiveness*” [11]. However, independently from definitions, the concept of AMS has progressively spread over the last few years and it is now accepted in promoting the correct and responsible use of antibiotics. Since there are several reasons for the phenomenon of increased antibiotic resistance, several different actions characterize AMS. Among these actions, there is the correct use of antimicrobials from a pharmacological point of view, which means excluding pharmacological factors that potentially ramp up the emergence of resistance, such as an inappropriate selection of the antimicrobial agent, errors in dosing and administration modality and, lastly, excessive duration of therapy [6]. More precisely, antimicrobials should be administered according to PK/PD principles. We suggest for example the use of simplified calculation of the AUC_0–24_ based on Begg [12] and Pai [13] methods. With the simplified method, it is possible to carry out TDM in a clinical setting because it is not necessary to draw several samples of blood.

When considering the appropriate selection and administration modality of antibiotics according to PK/PD parameters in ICU patients, the specific physio-pathological derangements typical of critically ill septic patients must be kept in mind [14]. Critically ill patients represent a non-homogeneous group of hospital patients who certainly differ from healthy volunteers or other hospitalized patients. These patients need aggressive medical interventions to counteract important pathophysiological changes, such as renal impairment, circulatory shock, altered vascular permeability and reduced albumin levels, to mention a few, which usually accompany sepsis [14]. Infections in these patients are characterized by significantly higher mortality rates, especially when sustained by Gram-negative strains [15]. Schematically, alterations in ICU septic patients can be classified into three categories; those due to sepsis, those due to organ dysfunction and those due to iatrogenic intervention. All of them affect antimicrobial therapy and must be considered when treating these patients.

Practically speaking there are “5Ds” that can help physicians to use antimicrobial therapy; they are: (1) Decision; (2) Drug; (3) Dose; (4) De-escalation; (5) Duration.

(1) Decision: Avoid using antibiotic therapy when it is not necessary. Prompt initiation of antibiotic therapy is an essential step to treat patients affected by septic shock [16], but too often, antimicrobials are given to patients not affected by bacterial diseases. The availability of more rapid microbiological diagnostic tools in the near future together with adequate patient stratification, will allow clinicians to wait before starting antimicrobial therapy, even in critically ill patients, thus restricting antimicrobial use [17].

(2) Drug: Choice of the right molecule, according to chemical-physical properties, spectrum of activity, mechanism of action, susceptibility, side effects and PK parameters, including tissue penetration. Scores aiming at more accurately targeting empiric therapy in settings with high resistance levels have been published even recently [18].

(3) Dosing: Dosing selection should follow PK/PD principles. PK describes a drug’s changing concentrations in the body compartments: PK knowledge enables the prediction of the time course of antimicrobial concentrations in the different tissues/fluids. The PK/PD characteristics of antibiotics describe the relationship between efficacy that is the in vitro susceptibility of a drug to a microorganism (usually expressed as minimal inhibitory concentration, MIC) and the in vivo exposure to the drug, which relies on the specific PK of the molecule and the dose. The “PK/PD target” is defined as the minimal PK/PD value that is associated with a high probability of successful treatment [19]. It is possible to share results derived from different in vivo models obtained even in different species since the antimicrobial target is the microorganism and not the host. Therefore, despite the obvious differences in PK between mice and patients, consideration of exposure relative to the MIC gives useful information to be used at bedside [20].

(4) De-escalation consists in switch from broad spectrum antibiotics to more targeted agents when culture results are available. A revaluation of the chosen antibiotic agents should be carried out after 48 h from initial prescription. Data about benefits of de-escalation even in setting with high percentage of multidrug resistant (MDR) pathogens are emerging [21], although this practice remains infrequently applied in ICU patients [22]. Both a Cochrane review [23] and meta-analysis [24] report that powerful evidences to support de-escalation of antibiotics in patients with sepsis, severe sepsis or septic shock are still lacking. 

(5) Duration: The optimization of treatment duration allows to avoid long administrations (10−14 days). A number of days commonly ranging from 5 to 8 days for several infections has been established [25]. This issue is more extensively analysed in a dedicated section.

Antibiotics are traditionally classified in two major groups: Time-dependent or concentration-dependent drugs [26]. Concentration-dependent drugs show maximal bactericidal activity when their concentrations are high in comparison to the MIC value (expressed by a high peak concentration (C_max_)/MIC)), even if these concentrations are maintained for a relatively short time. Therefore, these drugs are usually administered at high doses and with long intervals (i.e., one single daily dose or no more than two daily doses); these molecules usually show prolonged post-antibiotic effect (PAE). A typical example of these drugs is represented by aminoglycosides. Conversely, some molecules, such as beta-lactams, show time-dependent activity, and the concentrations of the free drug above the MIC for the specific pathogen at the infection site for a relatively prolonged time are necessary in order to optimize exposure. These drugs are characterized by a time-dependent killing. However, the maintenance of through concentrations ≥ 4 × MIC has been shown to prevent resistance [27]. More recently, a third group has been added to the classical ones. This group includes glycopeptides, oxazolidinones, colistin and fosfomycin and it is regulated by the AUC/MIC ratio, since it is related to both concentration- and time-dependent activity: for these antimicrobials the goal is to maximise the amount of drug administered daily [26]. Table 1 gives more specific data about the major classes of antimicrobials classified according to PK/PD principles.

Sepsis-associated PK changes greatly affect the penetration of antimicrobials in the infection sites. Consequently, in these clinical circumstances, the optimal treatment of bacterial infections usually requires a more aggressive dosing schedule. For time-dependent drugs, the application of prolonged or continuous infusion may be helpful, while for concentration-dependent antibiotics higher doses may be necessary. Although, tissue penetration of lipophilic agents is less affected than that of hydrophilic molecules (e.g., beta-lactams whose volume of distribution may be significantly increased leading to decreased plasma concentrations), they may achieve tissue concentrations higher than those in the plasma even in the critically ill patient. The frequency of pathogens with higher MICs remains higher in ICU patients compared to the community [2,28] and exposures in tissue risk being relatively inadequate. When MICs are high, near the resistance breakpoint, the PK/PD target cannot be achieved in ICU patients. Inadequate exposures favor the development of resistance, therefore the “optimal” therapy is the one which is chosen according to the likelihood of reaching the PK/PD target at infection sites. In the clinical setting, Monte Carlo simulation is extremely useful to help clinicians to predict the probability of success or failure of antibiotic therapy in the most complicated patients and/or in the most serious infections (see below).

## 3. PK/PD Issues and Antimicrobial Resistance

Optimization of antimicrobial use according to PK/PD principles has been studied extensively, and it has been shown to be associated with improved clinical outcome, even though the evidence from published studies is often flawed by methodological issues [29]. Optimization of PK/PD parameters in order to contain resistance has been less extensively investigated in clinical practice, although it sounds reasonable that, when microbiological eradication is achieved, resistance is contained.

There are several differences which must be considered when comparing results from clinical studies and experimental bacterial infection models, as previously reported [30] and some of them are summarized in Table 2.

Experimental models widely used to determine non-clinical in vivo PK/PD targets, include the neutropenic murine-thigh infection model, the lung infection model, the pyelonephritis model and the hollow-fiber infection model. Subsets of clinical isolates representative of multiple resistance mechanisms are also used to produce severe infections for instance in non-neutropenic mice [31]. Among all, hollow-fiber infection model may be very useful, as it allows us to overcome some limitations commonly associated with rodent PK/PD studies. It is possible to simulate a wide range of clinically relevant concentration-time profiles, to use an inoculum (10^8^ CFU/mL) as high as that occurring in severe infections, and finally to identify the antibiotic doses potentially capable of suppressing the amplification of the resistant mutant sub-populations [32].

Overall, it is important to investigate the containment of resistance, especially when considering several occurrences that strongly affect treatment results. Among them, the following play a pivotal role: i. Gram-negative strains harbor extended-spectrum beta-lactamase (ESBL) in more than 70% of cases in many countries [33]; ii. bloodstream infections due to MDR Gram-negative strains are associated with almost 3 times more mortality than infections caused by susceptible strains [34].

We report herein, data about PK/PD and resistance concerning the most important antimicrobial classes.

### 3.1. Beta-lactams 

Overall, beta-lactams are a class of antibiotics characterized by a very short half-life (from 0.5 h of several penicillins to about 4 h of some cephalosporins), with the exclusion of ceftriaxone which shares an elimination half-life of ≥8 h). Even though the most important parameter associated with efficacy (both clinical outcome and/or killing) for this class of antimicrobials is T>MIC, for suppression of emergence of resistance the C_min_/MIC parameter is also very important [35]. The value of both T>MIC and C_min_/MIC for suppressing resistance is not fixed for each beta-lactam, but depends on several factors, such as bacterial species, MIC values, duration of exposure and bacterial burden, that is the baseline CFU/mL utilized in the different studies, which can range from 5.5 × 10^5^ to 10^8^ CFU/mL, but is usually lower than that occurring in a clinical setting (see Table 2). For example, with a bacterial burden of 10^6^ CFU/mL, 100% T>MIC is sufficient for cephalosporins and penicillins to avoid the emergence of resistance when exposure lasts <72 h [36]. Felton et al. [37] examined piperacillin-tazobactam PK/PD index for suppressing emergence of resistance for both low (10^6^ CFU/mL) and high (10^8^ CFU/mL) *P. aeruginosa* burden. The high burden required a higher C_min_/MIC ratio than the low burden (ratio 4.6, and 3.4, respectively). Interestingly, when piperacillin-tazobactam was not given as a bolus but as prolonged infusion, C_min_/MIC ratios of 10.4 or 11.9 (for low, and high burden, respectively) were required. C_min_/MIC ratios >3.8 seem to be necessary for cefepime and ceftazidime [38]. Higher ratios (>6.2) have been reported for meropenem for preventing the development of resistance in *P. aeruginosa* [39]. The need of obtaining ratios higher than 6.2 has also been shown in a murine pneumonia model in which despite reaching the PK/PD target of fT > MIC of 40% and 100% survival, a suboptimal exposure caused selection of carbapenem-resistance in *P. aeruginosa* [40].

If it is important to preserve the activity of “old” molecules, it is much more important to preserve the new beta-lactam/beta-lactamases associations active against Gram-negative strains by using them judiciously. Few studies are available to describe dosing and selection of resistances for these associations. C_min_/MIC ratios of 1/0.5 or 2/1 have been described for ceftolozane/tazobactam to prevent the emergence of resistance, the target depending on the strain tested. For ceftolozane/tazobactam, in addition to the T>MIC or C_min_/MIC ratio, the AUC seems an important parameter to be considered in the prevention of resistance: with the 1–2 g dose, an AUC of 1032 mg/L∙h is obtained vs. an AUC of 456.2 mg/L∙h with the 0.5–1 g dose. Only higher AUCs are associated with C_min_ values which are capable of suppressing the emergence of resistance [41,42,43,44].

For the association meropenem/varborbactam, the free AUC_0–24_/MIC ratio is reported to be the parameter which better predicts both killing and resistance suppression: with AUC_0–24_/MIC values > 24, a 2-Log kill and suppression of the development of resistance is reported [45]. A ceftazidime-avibactam dose of 2.5 g q 8 h is suitable for treating urinary and abdominal infections. However, this dose may be sub-optimal for lung infections, where alterations in drug exposure can be the consequences of both the systemic changes typical of critically ill patients and of local factors occurring in the lung (such as hypoxic vasoconstriction). Indeed, while epithelial lining fluid (ELF) concentrations of ceftazidime/avibactam were 30% of those in plasma in healthy volunteers [46], similar exposures seem difficult to reach among severely ill patients [47]. This might expose to the risk of developing resistances.

The different values of T>MIC required to suppress resistance can partly be explained by the different pharmacodynamics of the different beta-lactams. Those molecules, like piperacillin, which act only on 1 penicillin-binding protein (PBP) have a low or almost absent bactericidal activity and are more dependent on the inoculum effect. Conversely, carbapenems, which inhibit more than 2 of the main PBPs, are not affected by the inoculum effect, and show a high killing activity. Cephalosporins such as ceftazidime, being capable of inhibiting no more than 2 PBPs, have intermediate behaviour [48].

Traditional modalities of beta-lactam administration are represented by bolus and by an intermittent infusion (30 min). However, in critically ill patients a continuous or extended infusion (≥3 h) is more and more used because it allows the persistence of higher beta-lactam concentrations above the MIC than intermittent infusions [49]. Whether such higher beta-lactam plasma concentrations translate in improved clinical outcomes is still debated [50], although the French Society of Pharmacology and Therapeutics (SFPT) and the French Society of Anaesthesia and Intensive Care Medicine (SFAR) provided in theirs recently Guidelines, a strong agreement in administering beta-lactam antibiotics by prolonged or continuous infusions in several settings of critically ill patients [51].

### 3.2. Colistin

This antimicrobial agent belongs to the polymixin family. The literature reports great variability of colistin PK in ICU patients, as expected by its highly hydrophilic properties [52,53]. The PK/PD parameter that best describes the success of therapy and probability of suppression of the emergence of resistance with colistin is the AUC/MIC ratio [52]. A loading dose of the prodrug colistimethate seems appropriate to avoid underexposure for treating severe infections. The rationale of this approach is based on the slow increase of plasma concentrations of formed colistin (from many hours to days) [54]. The inhaled therapy also appears to be useful, since nebulization helps to achieve high lung exposure. Inhaled 1M IU × 3 is associated with 6.7 mg/L in ELF 1 hr after administration, which is higher than plasma concentrations [55]. A study in the murine tight and lung infection model showed that *A. baumanni* developed resistance even for colistin exposure > 10 mg/L, which are concentrations much higher than those obtained with the classical clinical dose of 3M IU × 3 (which are about 2–3 mg/L) [56]. In a clinical setting, when a 9M IU loading dose was given, followed by 4.5M IU × 3, no resistance emerged in 127 ICU patients out of 130 [57]. However, loading doses are associated with an increased risk of renal failure [58]. In an in vitro model [59], no dose of colistin methanesulfonate or polymixin B was capable of suppressing the emergence of resistance [60]. Colistin, due to its high probability of inducing resistance, should never be given alone, even if administered at high doses.

### 3.3. Fluoroquinolones

For this group of antimicrobial drugs, the index associated with suppression of resistance is the AUC/MIC ratio, with values described between 300 and 1400. Therapy with fluoroquinolones in ICU patients has declined during the last few years, due to the rapid emergence of resistance after their use. In the management of community-acquired pneumonia, a combination of a beta-lactam antibiotic and a fluoroquinolone was associated with subsequent acquisition of MDR pathogen in 15% of patients, compared with 4% of patients who received a beta-lactam and macrolide combination [61].

### 3.4. Tetracyclines and glycylcyclines

For minocycline, an AUC/MIC between 20 and 25 is necessary to suppress resistance in *A*. *baumanni* [62]. Doses higher than those recommended (100 mg bid) are necessary for clinical cure and possibly to prevent resistance development [63].

The increasing incidence of MDR and extensively drug resistant (XDR) strains causing infections has recently renewed the use of tigecycline, often in combination chemotherapy. A recent metanalysis including 17 studies with 1104 pts has demonstrated that high doses of tigecycline (150–200 mg loading dose and then 75–100 mg BID) are more effective both microbiologically and clinically than standard doses without a significant impairment of safety [64].

### 3.5. Aminoglycosides

For this antimicrobial class, the PK/PD target for preventing the emergence of resistance depends on the specific molecule used, on the administration modality and on isolates. Consequently, when netilmicin is used, a C_max_/MIC ratio > 8 is necessary to suppress resistance against several Gram-negative strains such as *E. coli, P. aeruginosa* and *K. pneumoniae*. If amikacin is given once daily, a lower dose is necessary to suppress resistance than when it is administered twice (C_max_/MIC ratio 13 vs. C_max_/MIC ratio 20) [65].

### 3.6. Fosfomycin

This antimicrobial is usually prescribed for urinary infections in the community; in hospitals, its employment is reserved for the treatment of MDR bacteria as a 24 g i.v. daily dose. Resistance to fosfomycin is frequent (up to 20%) when treating infections other than those of the urinary tract [66]. The PK/PD parameter which is associated with less risk of emergence of resistance is the AUC/MIC ratio. In an in vitro model the daily human dose for ICU patients was simulated and this was sufficient for the suppression of resistance in *E. coli* with 1 mg/L MIC when an AUC_0–24_/MIC ratio of 3136 h-1 was obtained [67]. Fosfomycin should only be given in combination chemotherapy and at high daily doses.

## 4. Monte Carlo Simulation

Monte Carlo Simulation is a software able to perform virtual clinical trials by exploiting PK/PD observations from in vitro and in vivo models or from clinical data and thus extrapolating information that may predict exposure-response relationships that, in turn, can be translated to patients. It is based on few key factors such as a population PK model obtained from the study case series, descriptions of the effect of covariates that affect PK parameters, the description of the susceptibility of bacteria to the antibiotic and, finally, a PK/PD target associated with antibiotic efficacy. By integrating these factors, the system generates the outputs of the probability of target attainment (PTA) that describe the proportion of patients that will achieve a pre-specified PD target (PDT) for a MIC distribution. Therefore, these analyses are informative regarding dosing requirements associated with achieving PK/PD targets for pathogens with different MICs [68].

Monte Carlo Simulation may work at different degrees of complexity, the most challenging being the prediction of the effect of altered dosing strategies on the development of antibiotic resistance. Therefore, the optimization of antibiotic dosing in critically ill patients may benefit from this simulation model since the severe infections with which they may be affected require higher doses compared with the standardized ones. Also, Monte Carlo simulation may widely contribute to the definition of breakpoints of resistant pathogens as well as to their update [69,70].

Dose optimization in Gram-negative infections and the relevance of the use of Monte Carlo simulation in the dose optimization of beta-lactams regimens at the corresponding MICs has been recently reviewed [71].

Monte Carlo simulations have widely contributed to demonstrating the improvement of PK/PD outcomes for beta-lactams by prolonging their infusions in order to maximize the %fT > MIC. For instance, results of a recent review evaluating 39 studies based on the Monte Carlo simulation approach showed that prolonged infusion may enhance beta-lactams percent fT > MIC for unsusceptible *P. aeruginosa* (high MICs) or patients with altered PK profiles, including critically ill, patients [72].

The Monte Carlo simulation approach may also be used to study optimal drug exposure for combination therapy. As reported above, cefepime and tobramycin may be successfully combined for the treatment of *P. aeruginosa* in critically ill patients. In particular, Drusano et al. [73] used the Monte Carlo simulation approach to investigate the suppression of cefepime resistance by tobramycin for *P. aeruginosa* in patients with ventilator-associated pneumonia (VAP) and showed the benefit of this combination treatment for tobramycin MIC 1 mg/L. In fact, following 2 g of cefepime q8 h plus 7 mg/kg of tobramycin daily, cefepime targeted exposures were attained at a high rate even at a MIC value of 8 mg/liter (99.5%). The tobramycin target attainment was excellent for MIC values between 0.25 and 0.5 mg/L (100%), acceptable at 1 mg/L MIC (70%) and unacceptable for higher tobramycin MIC values.

Monte Carlo Simulation has been also successfully applied to investigate the highest probability of clinical success with the fluoroquinolone-based therapeutic options in ICU patients. On this issue, an interesting study was provided by Roberts et al. [74] who compared population PK in critically and not critically ill patients who received i.v. levofloxacin 500 or 750 mg q24 h. Monte Carlo simulations evidenced that, independently from the clinical conditions, patients with higher CrCl had lower PTA. Increasing doses resulted in increased PTA but the increase of dosing frequency had little effect.

Another example was provided by Lewis et al. [75]. PTAs of ciprofloxacin and levofloxacin were investigated by Monte Carlo Simulation in 5000 virtual critically ill patients undergoing prolonged intermittent renal replacement therapy (RRT). Results of this simulation study showed that conventional empiric single agent treatments did not meet desired PD targets (i.e., AUC_24h_:MIC of ≥125 for Gram-negative pathogens—reference pathogen *P. aeruginosa*), thus further supporting the usefulness of this approach especially in highly clinically complex populations in which real clinical PK trials are lacking. However, the actual use of quinolone-based combinations has been dramatically reduced due to the increased resistance of Gram-negative rods, and especially of ESBL producers [76].

Concerning polymixins, results of a recently published study evaluating drug resistant *A. baumannii* isolates showed by using Monte Carlo simulation that PTA for 300 mg of colistin base activity/day was <90% even for MIC ≥ 1 μg/mL [77]. Considering that colistin breakpoint is ≤2, the probability of underexposures that promote resistances is very high in the clinical setting, thus suggesting that colistin should be used in association.

## 5. Combination Therapy

Combination therapy has the biological plausibility to extend the spectrum of antimicrobials and possibly to have synergy and reduce the possibility of the emergence of resistance. Few studies, however, have addressed the effect of combination therapy toward the emergence of resistance. Beta-lactams and aminoglycosides represent a classical association that show synergistic activity against Gram-negative strains. Interestingly, low doses of tobramycin and cefepime (3 mg/kg and 563 mg given every 8 hr, respectively) suppressed resistance emergence of *P. aeruginosa* in a dynamic hollow fiber infection model (HFIM) [73]. Similarly, C_min_/MIC ratios < 1.7 vs. C_min_/MIC ratios > 6.2 are sufficient to suppress resistance in *P. aeruginosa* strains when meropenem is associated with tobramycin [39].

There is not much evidence-based clinical data supporting that beta-lactams/aminoglycosides association is capable of containing resistance. One possible explanation consists in the different dosing regimens used among studies included in a meta-analysis, and in the aminoglycoside multiple daily dosing regimens, rather than the once-daily dosing regimens used in in-vitro studies, which is more effective at reaching the C_max_/MIC target [35].

Despite potential beta-lactam antibiotic and fluoroquinolone synergistic activity [78,79], the same increased risk of emergence of ESBL-producing Enterobacterales exists following fluoroquinolone therapy (which is not seen with aminoglycoside administration), potentially due to mutagenesis mediated by fluoroquinolone exposure. The use of colistin in combination therapy with another agent may reduce the emergence of colistin resistance. Fosfomycin has been associated with polymixins to reduce resistances, with conflicting results depending on drugs, strains and models. For an exhaustive review see [35].

## 6. What Can Be Done for ICU Septic Patients in order to Apply PK/PD Principles: Focus on Renal Function, Discontinuation/Duration of Therapy and TDM

### 6.1. Renal Function

Renal function is frequently affected in patients requiring antibiotic therapy, especially if they are ICU patients [80]. Typically, changes in renal function are not stable during an ICU stay but undergo fluctuation, with a rapid and a great increase in the level of creatinine generally associated with sepsis. Therefore, it is important to adjust antimicrobial dosing according to a strict monitoring of kidney function and to balance the risk of toxicity with that of under-dosing [81]. There are 3 ways in which renal function can change in ICU septic patients: 1) impaired renal function/acute kidney injury (AKI), that can be treated with conservative therapy; 2) AKI treated with continuous renal replacement therapy (CRRT); 3) Augmented renal clearance (ARC).

#### 6.1.1. Impaired Renal Function/AKI Treated Conservatively

Most antimicrobials are excreted by the renal system and they should be given in a reduced dose in the case of stable renal insufficiency, in order to avoid toxic reactions linked to their accumulation. It has recently been shown, however, that this approach is not optimal for critical patients, because it seems to be associated with a trend of reduced efficacy [81]. In the RECLAIM trial, ceftazidime-avibactam was given in a reduced dose to patients with reduced creatinine clearance (CrCl) according to the manufacturer’s instructions, which are usually established on formulas such as the Cockcroft-Gault or the Modification of Diet in Renal Disease (MDRD). Patients with CrCl between 30 and 50 mL/min received ceftazidime-avibactam 1.25 g × 2 and had a lower percentage of microbiological and clinical cure (45.2% and 72%) than those with the same degree of renal impairment receiving meropenem 1 g × 2 (microbiological and clinical cure 74.3% and 88% respectively) [81]. Despite the fact that these studies were not statistically designed to make inferences in specific subgroups, and despite the fact that there are other potential explanations for the poorer outcome in patients with AKI, one possible explanation may be offered by PK/PD-related factors. It is likely that patients with AKI were underdosed. Moreover, in 67.9% of patients with initial CrCl < 50 mL/min, renal function improved within 72 h [82]. The Cockcroft-Gault or MDRD formulas, which have been validated in patients with a stable degree of renal impairment, are not suitable for critically ill patients. Bidell and Lodise [81] suggest that another method to measure creatinine in critically ill patients should be the “creatinine clearance equation” formula [83]. This formula takes into consideration not only the magnitude of creatinine rise and fall, but also the rapidity of the change.

#### 6.1.2. AKI Treated with Continuous Renal Replacement Therapy (CRRT)

CRRT can contribute to alteration of antimicrobial PK in critically ill patients, and the amount of change should be addressed with specific studies [84,85,86,87]. There are different modalities of CRRT and there is not one sole indication that should be adopted to optimize antibiotic dosing. However, CRRT can increase drug clearance and contribute to clinical failure and/or development of resistance, as recently shown in the CEFTABUSE study [88]. Patients with CRRT and pneumonia treated with ceftazidime-avibactam had a worse outcome. Moreover, Shields et al. [89] showed that in patients with pneumonia who received ceftazidime-avibactam there was an emergence of resistance in 10% of cases and that CRRT was an independent predictor of the development of resistance (*p* < 0.009). It is likely that these patients were under-dosed, due to the enhanced drug elimination because of renal supportive therapy. Generally, the effect of RRT on lipophilic drugs, which have higher Vd, is negligible.

#### 6.1.3. ARC

ARC usually refers to CrCl ≥ 130 mL/min/1.73 m^2^. This is typically observed in young patients with neurological trauma, burns or sepsis. The true prevalence of ARC is difficult to establish because the cut off varies among studies and because of the fluctuation of renal function in ICU patients [90]. However, in a prospective observational study of ICU patients, Udy et al. have reported that 65% of patients had ARC during the first week in the ICU [91].

The underlying mechanism seems to be an increased perfusion to the kidneys, as a consequence of sepsis-associated increased cardiac output and/or fluid resuscitation in critically ill patients.

Critical illness-associated systemic inflammatory response syndrome (SIRS), increases inflammatory mediators, changes in vascular permeability, temperature and alteration of cerebral autoregulation in cases of acute brain insult and ARC have been proposed as contributing/causal factors [90].

The degree of ARC is variable, sometimes the CrCl doubles [92]. Underdosing can expose patients to treatment failure and development of resistance. This can occur mostly with beta-lactam antibiotics, which are hydrophilic molecules primarily excreted through the kidneys, making their PK highly susceptible to the alterations seen in critical illness [93]. ARC entails PK/PD target attainment with intermittent dosing of piperacillin-tazobactam, for 100%fT > MIC target (not reached in 66% of the cases) using a MIC breakpoint of 16 mg/L [91]. Patients who failed to achieve 100%fT > MIC had significantly higher drug clearance (r = 0.58, *p* < 0.01), but no significant difference in Vd. Only 28.5% of patients developing ARC had a cumulative fraction of response at 100%fT > MIC. In a prospective observational study of ICU patients receiving meropenem 1 g every 8 hr or piperacillin/ tazobactam 4.5 g every 6 h [94], about half the patients had exposures lower than expected, a PK/PD target of 100%fT > MIC. Most of them had ARC, with median CrCl values of 165 (138–208) mL/min. In patients with ARC the PK/PD target of 50%fT > MIC was not reached in 37% of patients.

The effect of ARC on the possibility of reaching the PK/PD target also depends on the molecule in question. ARC seems to have no effects on meropenem 2g × 3 even for CrCl values of up to 250 mL/min, according to a study by Tamatsukuri et al. [95]. For linezolid, continuous infusion seems to allow higher probability of reaching an optimal PK/PD target than intermittent infusion [96].

Taken together, these data suggest that in ICU patients a reduction of dosing according to reduced renal function is not the optimal choice in the first 48 hr of treatment and that sometimes (i.e in ARC) it is necessary to increase antimicrobial dosing during the early phase of treatment [97]. The “creatinine clearance equation” formula [83] may offer a bedside tool to adjust dosing with renal function in a timely manner.

### 6.2. Discontinuation/Duration of Therapy

Another way to control the emergence of resistance is to reduce the time of antimicrobial administration, thus reducing antibiotic use and selective pressure. This can be achieved by using biomarkers.

There are some specific conditions which benefit from prolonged antimicrobial therapy, such as osteomyelitis or endocarditis. In other cases, it is important not to prolong antibiotic administration because it favors resistance. Clinical cure does not necessarily indicate microbial cure [14,98] because the immune system is capable of clearing residual bacteria; therefore, in a non-immunocompromised patient, shorter therapies are not associated with worse outcomes. However, this also depends on the type of patient/type of infection. It was the seminal paper of Chastre et al. [99] that, in a randomized controlled trial comparing 8 vs. 15 days of antimicrobial therapy for VAP, showed that patients receiving the shorter course had less-frequently recurring pulmonary infection with MDR pathogens (42.1% vs. 62%, short vs. long *p* < 0.05). However, when certain pathogens such as *P. aeruginosa* or *A. baumanni* are involved, or in certain sites (i.e., the lung), a tailored approach which integrates clinical judgment, biomarkers and microbiological results appears more prudent than a strict adhesion to a protocolled/fixed duration of therapy.

Since it is sometimes difficult to clinically distinguish the resolution of infection when an inflammatory response is still ongoing, biomarkers can help to stop antimicrobial therapy. Among the possible biomarkers available (IL6 and CPR, etc.), procalcitonin (PCT) has gained the most evidence [100]. PCT is a peptide precursor of the hormone calcitonin; the level of PCT rises above the limit of detection of clinical assays in response to several pro-inflammatory stimula, such as severe trauma surgery and infection. During renal failure PCT clearance is lowered; it usually shows a rapid decline in follow-up measurements when the patient recovers and its dynamic monitoring can be helpful. The application of PCT-guided therapy has been used mostly for respiratory infections, blood stream infections and sepsis. The first evidence that a PCT–guided cessation of antibiotic therapy was not inferior to standard care was offered by the PRORATA study [101]. Cochrane review of 2017 [102] has shown that PCT protocols help to decide whether to initiate or continue antibiotic therapy in acute respiratory failure based on initial PCT values. Also, PCT dynamic kinetics over time (80–90% decrease from peak) has been shown to reduce infection-associate adverse events, 28-day mortality and cost of hospitalization in sepsis [103]. The cut-offs should be modulated on the severity of the clinical setting, with cut-offs of 0.25 (0.1) ng/mL in respiratory infections and 0.5 ng/mL in sepsis [104]. PCT-guided therapy enables a 60–70% reduction in initiating antibiotic therapy for low-severity respiratory infection [105,106,107]. In cases of pneumonia, dynamic monitoring of PCT enables an earlier stop of antimicrobial therapy with reductions in the duration of antibiotic treatment ranging from 25% to 40% depending on the clinical picture. Although the reliability of PCT is maximal for bloodstream infections, PCT-guided therapy is useful for all septic patients. In a meta-analysis, including 1075 septic patients, there was a reduced antibiotic treatment duration (6 days versus 8 days) after PCT-guided therapy compared to standard care, without a worse outcome [108].

### 6.3. Bedside Therapeutic Drug Monitoring

The application of TDM enables physicians to individualize dosing. Although TDM has traditionally been designed as a process to reduce the risk of adverse events in patients receiving toxic drugs, at present its importance is being recognized in the optimization of therapeutic outcome, either in terms of cure or resistance suppression [109,110]. As stated above, the use of simplified calculation of the AUC_0–24_ methods [12,13,111] has been clinically applied to carry out TDM for some antibiotic classes since it is based on a small number of samples. TDM may be desirable for beta-lactams in order to optimize drug concentrations (4 × MIC) useful to prevent resistance [27].

As reported in the previous paragraphs, PK parameters of septic patients in the ICU may vary greatly, with a direct impact on PD parameters. More common sources of PK variability are an ARC due to increased renal blood flow and glomerular filtration that accelerates the elimination of renally cleared antibiotics or an augmented Vd due to leak syndrome. These, and other occurrences, that decrease the efficacy of antibiotics, further add complexity in these patients in which pathogens have higher MICs compared with those observed in other clinical settings [2]. On this basis, much evidence indicates that in critically ill patients antimicrobial dosing should be enhanced compared to non-critically ill patients, and that the PK/PD target should ideally be achieved from the first dose [112]. This is done to immediately kill bacterial subpopulations that are less susceptible to the specific antibiotic that otherwise will populate the site of infection. Therefore, in order to reduce the development of resistance and increase the chance of therapeutic success, dose adjustment should be carried out after the first dose. This approach substantially avoids the need to reach a steady state, which can delay the administration of the adjusted dose, and allows us to quickly reach the PD target through drug concentration measurements and dose optimization [109,113].

Therefore, with this aim, the use of TDM may also be applied to antibiotics traditionally not susceptible to TDM due to their low risk of side-effect development (e.g., beta-lactams) [110]. However, this procedure is generally rarely followed in clinics, probably due to several reasons, such as limited infrastructures and insufficient knowledge of PK [114,115]. Therefore, at present, specific TDM guidelines are substantially lacking. A relevant effort has recently been performed by SFPT and SFAR that in the framework of suggested guidelines for the optimization of the beta-lactam treatment in critically ill patients [51], have indicated some ‘optional’ recommendations regarding TDM. The term ‘optional’ has been introduced for all recommendation described in the document, due to the lack of important studies using mortality as main judgement criteria. The SFPT-SFAR suggested TDM guidelines concern, not only the patient’s conditions (i.e., ICU patients with expected beta-lactam PK variability and/or patients with clinical signs potentially related to beta-lactam toxicity; critically ill patients undergoing RRT), the PK parameters to be evaluated according to different modalities of administration (i.e., plasma trough concentration in case of intermittent administration, plasma steady-state concentration in case of continuous administration), the timing (24 to 48 h after the onset of treatment; after any change in dosage; in the event of significant change in the patient’s clinical condition), the kind of tissues (e.g., in the case of central nervous system infection, on blood and cerebrospinal fluid samples collected concomitantly), but also the modality of measurements (i.e., a validated chromatographic method). In relation to this last recommendation, it has to be considered that the method most commonly used for TDM involves immunological assaying that is less laborious compared with high-performance liquid chromatography (HPLC) technology even though less accurate [116].

From an informatic point of view, due to the amount of input data (covariates) and the complexity of the calculations (fitting, dosing) required for TDM analysis, a very high number of specific software has been developed [117,118]. Using TDM software to modify doses, it is possible to reach PK/PD targets, in order to prevent the emergence of drug resistance.

The results from the available studies, despite the above reported limitations, have demonstrated a substantial benefit of TDM in ICU patients affected by Gram-negative pathogens and represent useful tools, as well as the basis for future more powerful studies [119,120]. For example, a recent retrospective, single-center analysis of 484 patients with severe infections, sepsis and septic shock who received TDM-guided continuous infusion of piperacillin-tazobactam in ICU showed a PK-target attainment with an initial standard dose in 34.3% of patients. The successive TDM dose adjustments enhanced PK-target attainment in 62.4% of patients with the reduction of piperacillin-tazobactam clearance as well as of mortality, thus highlighting the potential useful role of TDM in controlling antibiotic resistance [121]. The results from the ongoing multicenter randomized controlled DOLPHIN trial that is investigating the effect of TDM of beta-lactams and fluoroquinolones on the outcome of critically ill patients will probably provide relevant insights on the role and clinical utility of TDM in this setting of patients. The study will compare active TDM versus non-TDM by evaluating 192 patients per group in 2 groups [122].

## 7. Conclusions

The correct use of antimicrobials can help to prevent the development and spreading of resistances. This is well-supported by pre-clinical data, while evidence in clinical settings is less available. Several reasons are responsible for the lack of evidence in the clinical setting, the difficulty in application of PK/PD principles to the single patient at bedside being one of the most important.

Since ICU patients are not homogeneous, and since the severity of their organ failure can rapidly change, their antibiotic therapy should be evaluated in terms of dosing and duration every day, according to their clinical picture, organ dysfunction (mainly kidney dysfunction) and biomarkers such as PCT. A simplified TDM—affordable at bedside—could be proposed to optimize therapy, with the aim, not only of avoiding toxicity, but also of avoiding underexposure which increases the risk of the emergence of resistance.

## Figures and Tables

**Table 1:** Pharmacokinetic/pharmacodynamic properties of selected antibiotics that correlate with efficacy.

	Pharmacodynamic Kill Characteristics		
	Time Dependent	Concentration Dependent	Concentration Dependent with Time Dependence
Antibiotic	Penicillins	Aminoglycosides	Fluoroquinolones
	Cephalosporins	Fluoroquinolones	Aminoglycosides
	Carbapenems	Fosfomycin	Fosfomycin
	Natural macrolides	Colistin	Colistin
	Clindamycin	Metronidazole	Glycopeptides
	Oxazolidinones	Daptomycin	Semisynthetic macrolides
		Metronidazole	Tetra- and Glycylcyclines
			Oxazolidinones
Optimal PK/PD index	T>MIC	C_max_/MIC	AUC_0–24_/MIC
Objective	Maximize duration of exposure	Maximize concentration	Maximize amount of drug exposure
Measures	Frequent administration or prolonged infusion dosing	Infrequent (once daily) administration of high doses	Administration of a high total daily dose

From [26], modified.

**Table 2:** Main differences between in vitro/in vivo experimental models and the clinical setting.

Parameter	In Vitro/in Vivo Experimental Models	Clinical Setting/Patients
Microorganism	Single	Multiple
Host	Rodent, rabbit (porcine)	Humans
Interaction with microbiome	Absent	Present/possible
Inoculum	Usually 10^4^–10^6^ CFU/mL	10^6^–10^9^ CFU/mL
Type of exposure	Controlled/static	Dynamic
Immune cells	Absent (in vitro), neutropenia (in vivo)	Present /decreased function
Clearance	Faster in rodents No specific models for ICU patients	High variability among patients, and in the same patient during evolution of the disease
